# Characterization of tamoxifen and 4-hydroxytamoxifen glucuronidation by human UGT1A4 variants

**DOI:** 10.1186/bcr1539

**Published:** 2006-08-02

**Authors:** Dongxiao Sun, Gang Chen, Ryan W Dellinger, Kimberly Duncan, Jia-Long Fang, Philip Lazarus

**Affiliations:** 1 Cancer Prevention and Control Program, Division of Population Sciences and Cancer Prevention, Department of Pharmacology, Penn State Cancer Institute, Penn State University College of Medicine, Hershey, Pennsylvania, USA; 2 Cancer Prevention and Control Program, Division of Population Sciences and Cancer Prevention, Department of Health Evaluation Sciences, Penn State Cancer Institute, Penn State University College of Medicine, Hershey, Pennsylvania, USA; 3 Division of Biochemical Toxicology, National Center for Toxicological Research, Jefferson, Arkansas, USA

## Abstract

**Introduction:**

Tamoxifen (TAM) is an antiestrogen widely used in the treatment and prevention of breast cancer in women. One of the major mechanisms of metabolism of TAM and one of its major active metabolites, 4-hydroxytamoxifen (4-OH-TAM), is via glucuronidation. In the present study, the glucuronidating activities of three common variant isoforms encoded by the human UDP-glucuronosyltransferase (UGT) 1A4 gene were examined against TAM, *trans*-4-OH-TAM and *cis*-4-OH-TAM.

**Methods:**

HPLC was used to detect glucuronide conjugates in microsomes from UGT1A4-overexpressing HK293 cells. The UGT1A4 wild-type cDNA was synthesized by RT-PCR using normal human liver total RNA. The UGT1A4^24Thr/48Leu ^and UGT1A4^24Pro/48Val ^variants were generated by site-directed mutagenesis of the pcDNA3.1/V5-His-TOPO plasmid expressing wild-type UGT1A4^24Pro/48Leu^. Levels of UGT1A4 expression in UGT-overexpressing cell lines were measured by western blot analysis.

**Results:**

Microsomes from wild-type UGT1A4^24Pro/48Leu^-overexpressing HK293 cells exhibited significant levels of activity against TAM, *trans*-4-OH-TAM and *cis*-4-OH-TAM, forming exclusively the tamoxifen quaternary ammonium glucuronide (TAM-*N*^+^-glucuronide) and the 4-hydroxytamoxifen quaternary ammonium glucuronides (*trans*-4-OH-TAM-*N*^+^-glucuronide and *cis*-4-OH-TAM-*N*^+^-glucuronide) with apparent *K*_m _values of 2.0 μM, 2.2 μM, and 2.1 μM, respectively. Higher glucuronidation activities were found by kinetic analysis for microsomes from the variant UGT1A4^24Pro/48Val^-overexpressing cell line as compared with microsomes from wild-type UGT1A4^24Pro/48Leu^-overexpressing cells against TAM and against both the *trans *and *cis *isomers of 4-OH-TAM. A significantly (*P *< 0.02) lower *K*_m _value (~1.6-fold to 1.8-fold) was observed for both 4-OH-TAM isomers, while a near-significant (*P *= 0.053) decrease in *K*_m _was observed for TAM for the UGT1A4^24Pro/48Val ^variant as compared with wild-type UGT1A4. The *V*_max_/*K*_m _ratio for the UGT1A4^24Pro/48Val ^variant was significantly (*P *≤ 0.005) higher than that observed for the wild-type UGT1A4 isoform for both the *trans *and *cis *isomers of 4-OH-TAM after normalization for UGT1A4 expression by western blotting. No significant effect on enzyme kinetics was observed for the UGT1A4^24Thr/48Leu ^variant against either isomer of 4-OH-TAM or with TAM.

**Conclusion:**

These data suggest that the UGT1A4 codon 48 Leu>Val polymorphism significantly alters glucuronidation rates against TAM and its active hydroxylated metabolites, and that this polymorphism may play an important role in individual pharmacological response to TAM therapy.

## Introduction

Tamoxifen (TAM) (1- [4-(2-dimethylaminoethoxy)-phenyl]-1,2-diphenylbut-1(Z)-ene) is a nonsteroidal antiestrogen commonly used for the treatment and prevention of estrogen-dependent breast cancer [1-4]. Adjuvant TAM treatment increases recurrence-free survival and overall survival in breast cancer patients with hormone receptor-positive tumors irrespective of their nodal status, menopausal status or age [5]. In addition to its antiestrogenic properties, which have been related to symptoms such as hot flashes, vaginal bleeding and pruritus vulvae [2,6], TAM also has partial estrogen-agonistic effects that may be linked to reduced risk for ischemic heart disease and osteoporosis [7,8], but may also increase the risk for endometrial cancer [9,10] and venous thromboembolism [11]. Although TAM is generally well tolerated, there is significant interindividual variability in the clinical efficacy of TAM as well as in the toxicities of TAM. For instance, about 30% of patients acquire TAM resistance and relapse [12]. In addition, the relative risk of endometrial cancers in patients treated with TAM is estimated to be twofold to threefold that of controls, the risk increasing with both the duration of and cumulative dose of TAM treatment [10,13-15].

The mechanisms underlying variability in response to TAM and to TAM-related toxicities remain obscure. Since there is compelling evidence that TAM is converted to antiestrogenic metabolites more potent than TAM itself, one hypothesis is that altered patterns of metabolism of TAM and/or its primary metabolites might contribute to this interindividual variability.

TAM is activated predominantly via cytochrome P450-mediated pathways into several metabolites after oral administration, including the hydroxylated TAM metabolites, 4-hydroxytamoxifen (4-OH-TAM) and 4-hydroxy-*N*-desmethyl-TAM (endoxifen). Since both *trans*-4-OH-TAM and endoxifen exhibit up to 100-fold the levels of antiestrogenic activity compared with TAM and other TAM metabolites [16-21], it is thought they may be the major contributors to TAM's antiestrogenic properties. While *cis*-4-OH-TAM is thought to be primarily an estrogen agonist, this isomer exhibits significant antiestrogenic activity *in vitro *when in the presence of estradiol [22-24].

An important route of elimination and detoxification of TAM and its metabolites is via glucuronidation. TAM is excreted predominantly through the bile, a process largely facilitated by TAM conjugation to glucuronic acid during the glucuronidation process [25], and TAM glucuronides have been identified in the urine of TAM-treated patients [26]. Most of the 4-OH-TAM in the bile of TAM-treated patients was found as a glucuronide conjugate [25,27]. The fact that TAM metabolites are found in their unconjugated form in feces is probably due to β-glucuronidase-catalyzed deglucuronidation within the microflora that colonize within the small intestine [25]. TAM glucuronide conjugates have been identified in the serum of TAM-treated patients [25,27], and it has been suggested that glucuronidation within target tissues such as the adipose tissue of the breast may also be important in terms of TAM metabolism and overall TAM activity [28].

One of the major UDP-glucuronosyltransferases (UGTs) involved in the glucuronidation of TAM and its metabolites is the hepatic enzyme UGT1A4 [29,30], which catalyzes the formation of a quarternary ammonium-linked glucuronide with TAM's *N*, *N*-dimethylaminoalkyl side chain [30]. This pattern of ammonium-linked glucuronidation is consistent with UGT1A4's glucuronidation activity against primary, secondary and tertiary amines present in a variety of carcinogenic compounds, androgens, progestins and plant steroids [31-34].

Two unlinked missense polymorphisms were identified at codon 24 (Pro>Thr) and codon 48 (Leu>Val) within the UGT1A4 gene [35,36]. The prevalence of both variant UGT1A4^24Thr/48Leu ^and UGT1A4^24Pro/48Val ^alleles approached 0.10 in Caucasian populations [35,36]. The codon 24 polymorphism was linked to altered glucuronidation activity against the tobacco-specific nitrosamine, 4(methylnitrosamino)1(3pyridyl)1butanol [36], while the UGT1A4^48Val ^variant was associated with decreased rates of glucuronidation after transient transfection into cell lines *in vitro *[35].

The aim of the present study was to characterize UGT1A4-induced glucuronidation of TAM, *trans*-4-OH-TAM and *cis*-4-OH-TAM, and to examine whether missense variants in the UGT1A4 gene alter activity against TAM and its hydroxylated metabolites *in vitro*.

## Materials and methods

### Chemicals and materials

UDP-glucuronic acid (UDPGA), *trans*-TAM, *trans*-4-OH-TAM (98% pure), *trans*-4-OH-TAM:*cis*-4-OH-TAM (70:30% ratio), alamethicin, β-glucuronidase, anticalnexin antibody and bovine serum albumin were purchased from Sigma-Aldrich (St Louis, MO, USA). HPLC-grade ammonium acetate and acetonitrile were purchased from Fisher Scientific (Pittsburgh, PA, USA) and were used after filtration. DMEM, Dulbecco's phosphate-buffered saline (minus calcium chloride and magnesium chloride), fetal bovine serum, penicillin-streptomycin and geneticin (G418) were purchased from Gibco (Grand Island, NY, USA).

The Platinum^® ^*Pfx *DNA polymerase and the pcDNA3.1/V5-His-TOPO mammalian expression vector were obtained from Invitrogen (Carlsbad, CA, USA), while the restriction enzymes Dpn*I *and Stu*I *were purchased from New England Biolabs (Beverly, MA, USA). The BCA protein assay kit was purchased from Pierce (Rockford, IL, USA) while the QIAEX^® ^II gel extraction kit was purchased from Qiagen (Valencia, CA, USA). The human UGT1A western blotting kit and anti-UGT1A antibody were purchased from Gentest (Woburn, MA, USA). All other chemicals used were purchased from Fisher Scientific (Pittsburgh, PA, USA) unless otherwise specified.

### Tissues

A description of the normal human liver tissue specimens used for these studies was presented previously [36]. Briefly, tissues were quick-frozen at -70°C within 2 hours postsurgery. Liver microsomes were prepared through differential centrifugation as previously described [37] and were stored (10–20 mg protein/ml) at -70°C. Microsomal protein concentrations were measured using the BCA assay (Pierce). All protocols involving the analysis of tissue specimens were approved by the institutional review board at the Penn State College of Medicine and in accordance with assurances filed with and approved by the United States Department of Health and Human Services.

### UGT1A4 cloning

The UGT1A4 wild-type cDNA was synthesized by RT-PCR using normal human liver total RNA and was inserted into the pcDNA3.1/V5-His-TOPO plasmid. The sense and antisense primers used for RT-PCR were UGT1A4s1 (5'-ACAGTCAGCTGTCGGTGGC-3', corresponding to -29 to -11 relative to the UGT1A4 translation start site; GenBank accession number NM007120) and UGT1A4as1 (5'-ATTTTACCTTATTTCCCACCC-3', corresponding to +1611 to +1631 relative to the UGT1A4 translation start site), respectively.

Incubations were performed in a GeneAmp 9700 thermocycler (Applied Biosystems, Foster City, CA, USA) as follows: one cycle at 94°C for 2 minutes, 41 cycles at 94°C for 30 seconds, at 55°C for 30 seconds and at 72°C for 2 minutes, followed by a final cycle of 7 minutes at 72°C. The PCR product (1662 base pairs) was purified after electrophoresis in 1.5% agarose using the QIAEX^® ^II gel extraction kit (Qiagen), and was subsequently subcloned into the pcDNA3.1/V5-His-TOPO mammalian expression vector using standard methodologies.

Confirmation of insert orientation was performed by restriction enzyme digestion, and UGT1A4 sequences were confirmed by dideoxy sequencing of the entire PCR-amplified UGT1A4 cDNA product (performed at the Molecular Biology Core Facility at Penn State University College of Medicine) using two vector primers (T7 and BGH; IDT, Coralville, IA, USA) and one internal sense primer (UGT1A4s2 – 5'-GAAGGAATTTGATCGCGTTAC-3', corresponding to nucleotides +258 to +278 relative to the UGT1A4 translation start site). The cloned UGT1A4 insert was compared with that described in GenBank and was confirmed to be 100% homologous to the wild-type UGT1A4^24Pro/48Leu ^sequence (accession number NM007120).

### Generation of UGT1A4 variants, overexpressing cell lines and cell microsome preparation

The UGT1A4^24Thr/48Leu ^and UGT1A4^24Pro/48Val ^variants were generated by site-directed mutagenesis of the pcDNA3.1/V5-His-TOPO plasmid expressing wild-type UGT1A4^24Pro/48Leu^. The primers used to change codon 24 from Pro to Thr were UGT1A4c24F (5'-CAGTGTCCAG**A**CCTGGGCTGAGAGTG-3') and UGT1A4c24R (5'-CACTCTCAGCCCAGG**T**CTGGACACTG-3'), both primers corresponding to nucleotides +60 to +85 relative to the UGT1A4 translation start site (mutated base indicated in bold). The primers used to change codon 48 from Leu to Val were UGT1A4c48F (5'-CTCAGCATGCGGGAGGCC**G**TGCGGGAGCTCCATGC-3') and UGT1A4c48R (5'-GCATGGAGCTCCCGCA**C**GGCCTCCCGCATGCTGAG-3'), both primers corresponding to nucleotides +124 to +158 relative to the UGT1A4 translation start site.

The products were amplified by PCR using 5 U/ml *Pfx *polymerase, 1 × *Pfx *buffer, 2 × enhancer, 10 μM dNTPs, 500 ng template, 1 mM MgSO_4 _and 20 μM of each primer in a BioRad MyCycler (Hercules, CA, USA) with an initial denaturation at 95°C for 2 minutes, followed by 25 cycles at 95°C for 30 seconds, at 65°C for 30 seconds and at 68°C for 18 minutes. Following amplification, 20 U Dpn *I*restriction enzyme was added to each reaction and incubated for 1 hour at 37°C to specifically digest the wild-type template DNA. The remaining PCR product was then transformed into competent DH5α *Escherichia coli*, individual colonies were isolated, and subsequent plasmid DNA minipreps were screened for the UGT1A4^24Thr/48Leu ^and UGT1A4^24Pro/48Val ^variants using HPY188III or Stu*I*, respectively. The UGT1A4^24Thr/48Leu ^and UGT1A4^24Pro/48Val ^cDNA sequences were confirmed by direct dideoxy sequencing using the same primers used for wild-type UGT1A4 analysis described earlier.

Human embryonic kidney fibroblast HEK293 cell lines overexpressing wild-type or variant UGT1A4 were generated by standard electroporation techniques in the Bio-Rad GenePulser Xcell using 10 μg pcDNA3.1/V5-His-TOPO/UGT1A4 plasmid DNA with 5 × 10^6 ^HEK293 cells (in 0.5 ml) in serum-free DMEM media, with electroporation at 250 V and 1000 μF. Following transfection, HK293 cells were grown in 5% CO_2 _to 80% confluence in DMEM supplemented with 4.5 mM glucose, 10 mM HEPES, 10% fetal bovine serum, 100 U/ml penicillin, 100 μg/ml streptomycin and geneticin (700 μg/ml medium) for the selection of geneticin-resistant clones, with selection medium changed every 3–4 days.

Individual UGT1A4-overexpressing cell colonies were selected and monitored for UGT1A4 expression via western blotting analysis (described in the next section). Cell homogenates were prepared by resuspending pelleted cells in Trisbuffered saline (25 mM Tris base, 138 mM NaCl, 2.7 mM KCl, pH 7.4) and subjecting them to three rounds of freeze-thaw prior to gentle homogenization. Microsomal fractions were prepared by centrifugation of whole cell homogenate at 10,500 rpm (9,000 × *g*) for 30 minutes at 4°C; the supernatant was collected and subsequently centrifuged at 33,500 rpm (105,000 × *g*) for 60 minutes at 4°C in a SW-55 Ti rotor (Beckman, Palo Alto, CA, USA). Pellets were collected by suspension in Tris-buffered saline (25 mM Tris base, 138 mM NaCl, 2.7 mM KCl, pH 7.4) and were stored at -80°C in 100 μl aliquots. Total microsomal protein concentrations were measured using the BCA protein assay (Pierce).

### Western blot analysis

Levels of UGT1A4 expression in UGT-overexpressing cell lines were measured by western blot analysis using the anti-UGT1A antibody in a 1:5,000 dilution as per the manufacturer's instructions (Gentest), while calnexin protein levels were assayed using a 1:5,000 dilution of the monoclonal anticalnexin antibody (after stripping the UGT1A4 antibody of the same western blot using standard techniques). UGT1A4 was detected by chemiluminescence using the SuperSignal West Dura Extended Duration Substrate (Pierce Biotechnology, Inc., Rockford, IL, USA). Secondary antibodies supplied with the Dura ECL kit (anti-rabbit and anti-mouse) were used at 1:3,000. UGT1A4 levels were quantified against a known amount of human UGT1A protein (200–300 ng, supplied in the western blotting kit; Gentest) by densitometric analysis of X-ray film exposures (1 s–1 min exposures) of western blots using a GS-800 densitometer with Quantity One software (Bio-Rad).

UGT1A4 protein levels were calculated against known titrated quantities of UGT1A standard, with quantification made relative to the levels of calnexin observed in each lane (quantified by densitometric analysis of western blots as already described). Determinations of aglycone-glucuronide formation in UGT1A4-overexpressing cell lines were calculated relative to the levels of UGT1A4 expression in the respective cell lines. X-ray film bands were always below densitometer saturation levels as indicated by the densitometer software. Densitometric results were always consistent irrespective of the exposure time. Western blot analysis and subsequent densitometric analysis were performed in triplicate on three separate occasions, using the same UGT1A4-containing cell homogenates used for activity assays, with relative UGT1A4 protein levels expressed as the mean of these experiments.

### Glucuronidation assays

Glucuronidation activities of microsomes from human UGT1A4-overexpressing HEK293 cells toward *trans*-TAM, *trans*-4-OH-TAM and *cis*-4-OH-TAM were performed after an initial incubation of microsomal protein (5–25 μg) with alamethicin (50 μg/mg protein) for 15 minutes in an ice bath. Glucuronidation reactions were then performed in a final reaction volume of 100–500 μl at 37°C in 50 mM Tris–HCl (pH 7.4), 10 mM MgCl_2_, 4 mM UDPGA and 1–6 μM *trans*-TAM or *trans*-4-OH-TAM, and 1–15 μM *cis*-4-OH-TAM. Reactions were terminated by the addition of 95–495 μl cold methanol on ice. Five microliters of propranolol (2.5 μg/ml) was added to the final reactions, the mixtures were centrifuged for 10 minutes at 4°C at 16,100 × *g*, the supernatants were collected and evaporated, and the resulting dried sample was resuspended in 200 μl of 50% methanol. Assays with human liver microsomes were identical except that 10 μg microsomal protein was used in glucuronidation assays.

Samples (100 μl) were analyzed for glucuronidated TAM or for TAM metabolites by HPLC using a Beckman Coulter System Gold 126 Solvent Module HPLC system (Fullerton, CA, USA) equipped with an automatic injector (model 508) and a UV detector operated at 254 nm (model 168). HPLC was performed using a 3 μ Luna C_18 _analytical column (4.6 mm × 150 mm; Phenomenex, Torrance, CA, USA) in series with a 5 μ Aquasil C_18 _guard column (10 mm × 4 mm; Thermo Hypersil-Keystone, Bellefonte, PA, USA). The gradient elution conditions for assays with *trans*-4-OH-TAM or *cis*-4-OH-TAM were as follows: starting with 75% buffer A (100 mM ammonium acetate, pH 5.0) and 25% acetonitrile (5 min), a subsequent linear gradient to 75% acetonitrile (25% buffer A) over 25 min was performed and then maintained at 75% acetonitrile for 10 minutes. The flow rate was 0.5 ml/min. For assays with TAM, the linear gradient was from 70% buffer A (30% acetonitrile) to 90% acetonitrile (10% buffer A) over 30 minutes.

The amount of *N*^+^-glucuronide formed was calculated based on the ratio of the peak area of the *N*^+^-glucuronide versus that observed for the internal standard, propranolol. Tamoxifen quaternary ammonium glucuronide (TAM-*N*^+^-glucuronide), the 4-hydroxytamoxifen quaternary ammonium glucuronides (*trans*-4-OH-TAM-*N*^+^-glucuronide and *cis*-4-OH-TAM-*N*^+^-glucuronide), and *trans*-4-OH-TAM-*O*-glucuronide and *cis*-4-OH-TAM-*O*-glucuronide were confirmed by 1 M NaOH hydrolysis and sensitivity to β-glucuronidase as previously described [34]. As controls, glucuronidation assays were regularly performed using human liver microsomes (as a positive control for glucuronidation activity) and untransfected HK293 cell homogenate protein (as a negative control for glucuronidation activity) as previously described [34,38]. Experiments were always performed in triplicate in independent assays.

### Liquid chromatography–mass spectrometry identification of 4-OH-TAM-N^+^-glucuronides

The predicted *trans*-4-OH-TAM-*N*^+^-glucuronide and *cis*-4-OH-TAM-*N*^+^-glucuronide were collected after separation by HPLC as already described and were identified by liquid chromatography–mass spectrometry (LC–MS) using a Shimadzu LC-MS 2010 EV system (Shimadzu, Tokyo, Japan). The *trans*-4-OH-TAM-*N*^+^-glucuronide and *cis*-4-OH-TAM-*N*^+^-glucuronide were loaded onto a Shimadzu reverse-phase column (Shimadzu C18, 4.6 mm × 50 mm) and were analyzed at a flow rate of 0.2 ml/min by applying a linear mobile phase gradient from 10% to 80% (v/v) methanol/H_2_O over 30 minutes. An electrospray voltage of 1.5 kV was applied using a positive mode.

### Statistical analysis

The Student *t *test (two-sided) was used for comparing the rates and the kinetic values of glucuronide formation for the UGT1A4^24Pro/48Leu^, UGT1A4^24Thr/48Leu ^and UGT1A4^24Pro/48Val ^variant isoforms against the different substrates examined in this study. Kinetic constants were determined using Graphpad Prism4 software (GraphPad Software, San Diego, CA, USA).

## Results

### Characterization of 4-HO-TAM glucuronides in microsomes from human liver and UGT1A4-overexpressing cells

**Figure 1:**
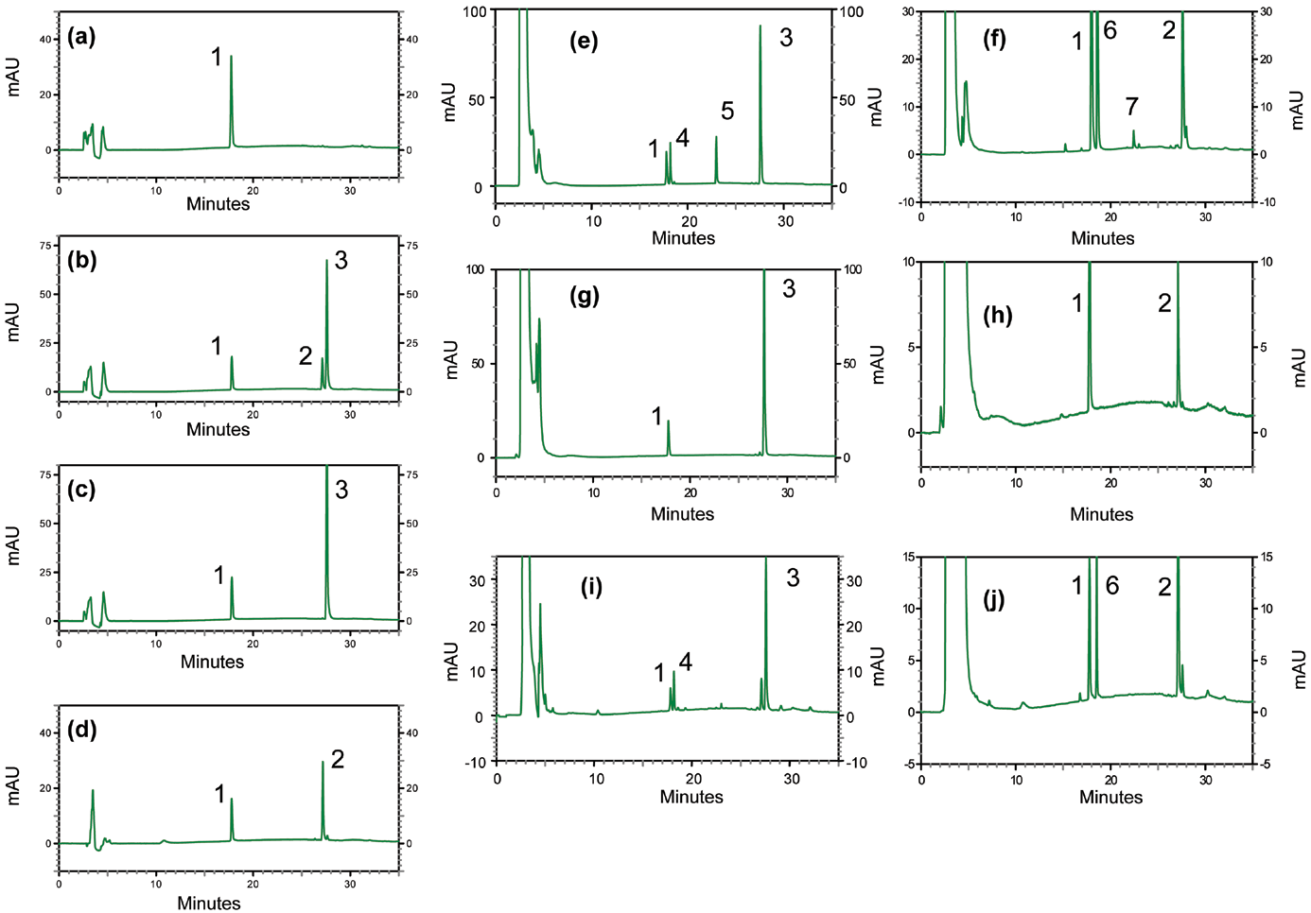
HPLC analysis of 4-hydroxytamoxifen glucuronides formed by human liver microsomes. Human liver microsomes (HLM; 10 μg protein) were incubated at 37°C for 30 min with 25 μM *trans*-4-hydroxytamoxifen (*trans*-4-OH-TAM) or 15 μM *cis*-4-OH-TAM and 4 mM UDP-glucuronic acid prior to analysis by HPLC as described in Materials and methods. Propranolol was added to all assays postincubation as an internal standard for HPLC. **(a) **No substrate, internal standard (propranolol) only; **(b) **nonincubated 70:30 mixture of *trans*-4-OH-TAM:*cis*-4-OH-TAM (Sigma); **(c) **nonincubated 98% pure *trans*-4-OH-TAM (Sigma); **(d) **nonincubated *cis*-4-OH-TAM purified from the 70:30 mixture shown in (b); **(e) ***trans*-4-OH-TAM incubated with HLM; **(f) ***cis*-4-OH-TAM incubated with HLM; **(g) ***trans*-4-OH-TAM incubated with HLM and β-glucuronidase; **(h) ***cis*-4-OH-TAM incubated with HLM and β-glucuronidase; **(i) ***trans*-4-OH-TAM incubated with HLM and 1 N NaOH; **(j) ***cis*-4-OH-TAM incubated with HLM and 1 N NaOH. Peak 1, propranolol; peak 2, *cis*-4-OH-TAM; peak 3, *trans*-4-OH-TAM; peak 4, *trans*-TAM-4-*O*-glucuronide (predicted); peak 5, *trans*-4-OH-TAM-*N*^+^-glucuronide (predicted); peak 6, *cis*-TAM-4-*O*-glucuronide (predicted); peak 7, *cis*-4-OH-TAM-*N*^+^-glucuronide (predicted). mAU, milli-absorbance units.

While *N*^+^-glucuronide was shown to be the only glucuronide formed with TAM in human liver microsomes and UGT1A4-overexpressing baculosomes [30], 4-OH-TAM *O*-glucuronides and *N*^+^-glucuronides were formed by human liver microsomes. As shown in Figure 1, a HPLC assay was developed to efficiently separate TAM and TAM metabolites from their glucuronide conjugates.

Using propranolol as an internal standard (Figure 1a), the *cis *and *trans *isomers of 4-OH-TAM eluted at retention times of 27.1 minutes and 27.6 minutes, respectively, as shown by HPLC of a 70:30 mix of *trans*-4-OH-TAM:*cis*-4-OH-TAM (Sigma) (Figure 1b), of 98% pure *trans*-4-OH-TAM (Sigma) (Figure 1c), and of *cis*-4-OH-TAM purified from the 70:30 *trans*-4-OH-TAM:*cis*-4-OH-TAM mix (Sigma) (Figure 1d). In assays with human liver microsomes, two 4-OH-TAM glucuronide peaks with retention times of 18.2 minutes (peak 4) and 23.0 minutes (peak 5) were observed in assays with *trans*-4-OH-TAM (Figure 1e), and two 4-OH-TAM glucuronide peaks with retention times of 18.6 minutes (peak 6) and 22.5 minutes (peak 7) were observed in assays with *cis*-4-OH-TAM (Figure 1f). Peaks 4–7 were sensitive to treatment with β-glucuronidase in glucuronidation assays with either *trans*-4-OH-TAM (Figure 1g) or *cis*-4-OH-TAM (Figure 1h). Only peaks 5 and 7 were sensitive to treatment with alkali after glucuronidation assays with either *trans*-4-OH-TAM (Figure 1i) or *cis*-4-OH-TAM (Figure 1j).

**Figure 2:**
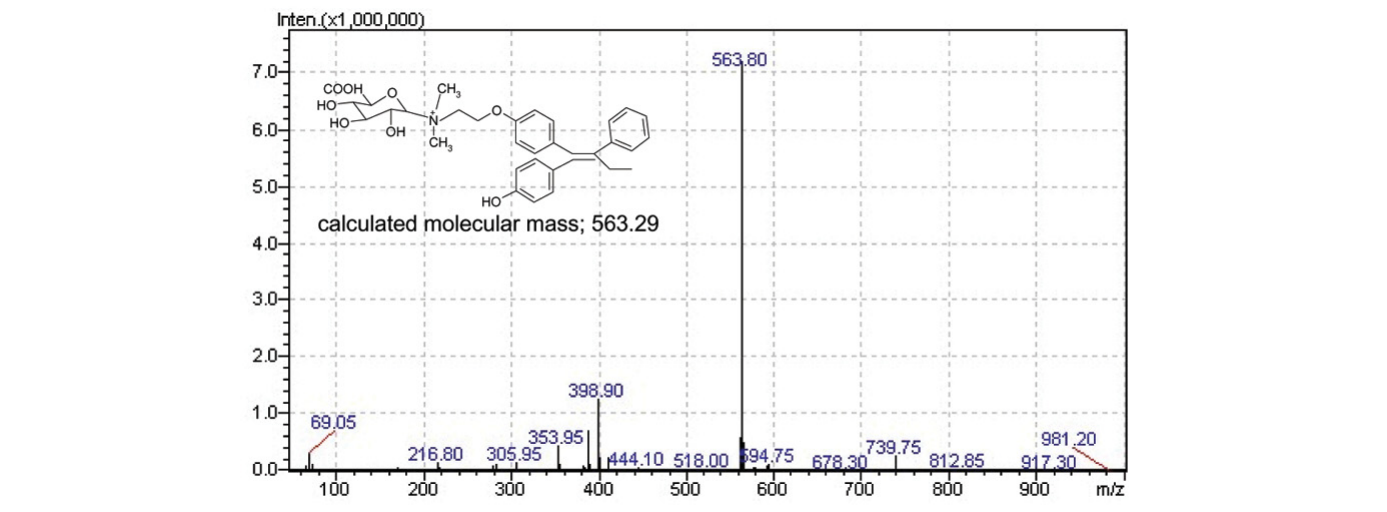
Representative mass spectra of *trans*-4-hydroxytamoxifen quaternary ammonium glucuronide formed by human liver microsomes. The predicted *trans*-4-hydroxytamoxifen quaternary ammonium glucuronide (*trans*-4-OH-TAM-*N*^+^-glucuronide) was collected after separation by HPLC and identified by liquid chromatography–mass spectrometry using a Shimadzu LC-MS 2010 EV system. *trans*-4-OH-TAM-*N*^+^-glucuronide was loaded onto a C18 Shimadzu reverse-phase column and analyzed at a flow rate of 0.2 ml/min by applying a linear mobile phase gradient from 10% to 80% (v/v) methanol/H_2_O over 30 min. An electrospray voltage of 1.5 kV was applied using a positive mode.

This pattern is similar to that observed for *N*-glucuronide versus *O*-glucuronide deconjugation for other compounds [32] – suggesting that peaks 4 and 6 corresponded to *trans*-TAM-4-*O*-glucuronide and *cis*-TAM-4-*O*-glucuronide, and that peaks 5 and 7 corresponded to *trans*-4-OH-TAM-*N*^+^-glucuronide and *cis*-4-OH-TAM-*N*^+^-glucuronide in assays with either *trans*-4-OH-TAM or *cis*-4-OH-TAM, respectively. The products corresponding to predicted *trans*-OH-TAM-*N*^+^-glucuronide and *cis*-4-OH-TAM-*N*^+^-glucuronide were analyzed using LC with electrospray MS detection (Figure 2). The mass spectrum for the *trans*-4-OH-TAM-*N*^+^-glucuronide showed a clear [M^+^] ion at *m/z *563.80 (calculated molecular weight, 563.29); a virtually identical pattern was observed for *cis*-4-OH-TAM-*N*^+^-glucuronide, with a clear [M^+^] ion at *m/z *563.70 (data not shown). These data suggest that the glucuronides derived from both isomers were in fact monoglucuronides.

**Figure 3:**
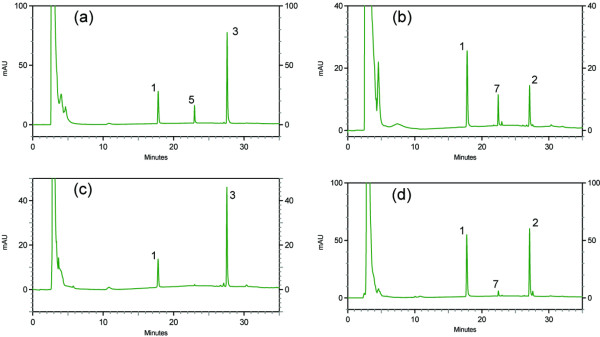
HPLC analysis of 4-hydroxytamoxifen glucuronides formed by wild-type UGT1A4-overexpressing cell microsomes. UGT1A4^24Pro/48Leu^-overexpressing cell microsomes (10 μg protein) were incubated at 37°C for 30 min with 25 μM *trans*-4-hydroxytamoxifen (*trans*-4-OH-TAM) or 15 μM *cis*-4-OH-TAM and 4 mM UDP-glucuronic acid prior to analysis by HPLC as described in Materials and methods. Propranolol was added to all assays postincubation as an internal standard for HPLC. **(a) ***trans*-4-OH-TAM incubated with UGT1A4-overexpressing microsomes; **(b) ***cis*-4-OH-TAM incubated with UGT1A4-overexpressing microsomes; **(c) ***trans*-4-OH-TAM incubated with UGT1A4-overexpressing microsomes and 1 N NaOH; **(d) ***cis*-4-OH-TAM incubated with HLM and 1 N NaOH. Peak 1, propranolol; peak 2, *cis*-4-OH-TAM; peak 3, *trans*-4-OH-TAM; peak 5, *trans*-4-OH-TAM-*N*^+^-glucuonide; peak 7, *cis*-4-OH-TAM-*N*^+^-glucuonide (peak numbers consistent with those described for Figure 1). mAU, milli-absorbance units.

In assays with microsomes prepared from wild-type UGT1A4^24Pro/48Leu^-overexpressing cells, significant glucuronidating activities were observed against both *trans*-4-OH-TAM and *cis*-4-OH-TAM (Figure 3). Single glucuronide peaks were observed in assays with either substrate (Figure 3a,b), with these peaks corresponding to the retention times of peaks 5 and 7 in glucuronidation assays with human liver microsomes (see Figure 1). These peaks were alkali sensitive (Figure 3c,d) and were sensitive to treatment with β-glucuronidase (results not shown), suggesting that these glucuronides corresponded to 4-OH-TAM-*N*^+^-glucuronide. Predicted UGT1A4-induced monoglucuronides were confirmed by LC–MS for both *trans*-4-OH-TAM and *cis*-4-OH-TAM (results not shown). No TAM-4-*O*-glucuronide formation was observed in assays with either the *trans *or *cis *isomer of 4-OH-TAM with UGT1A4-overexpressing cell microsomes, and no glucuronidation activity was observed for untransfected HK293 cell homogenates for any substrate examined in this study (results not shown).

**Figure 4:**
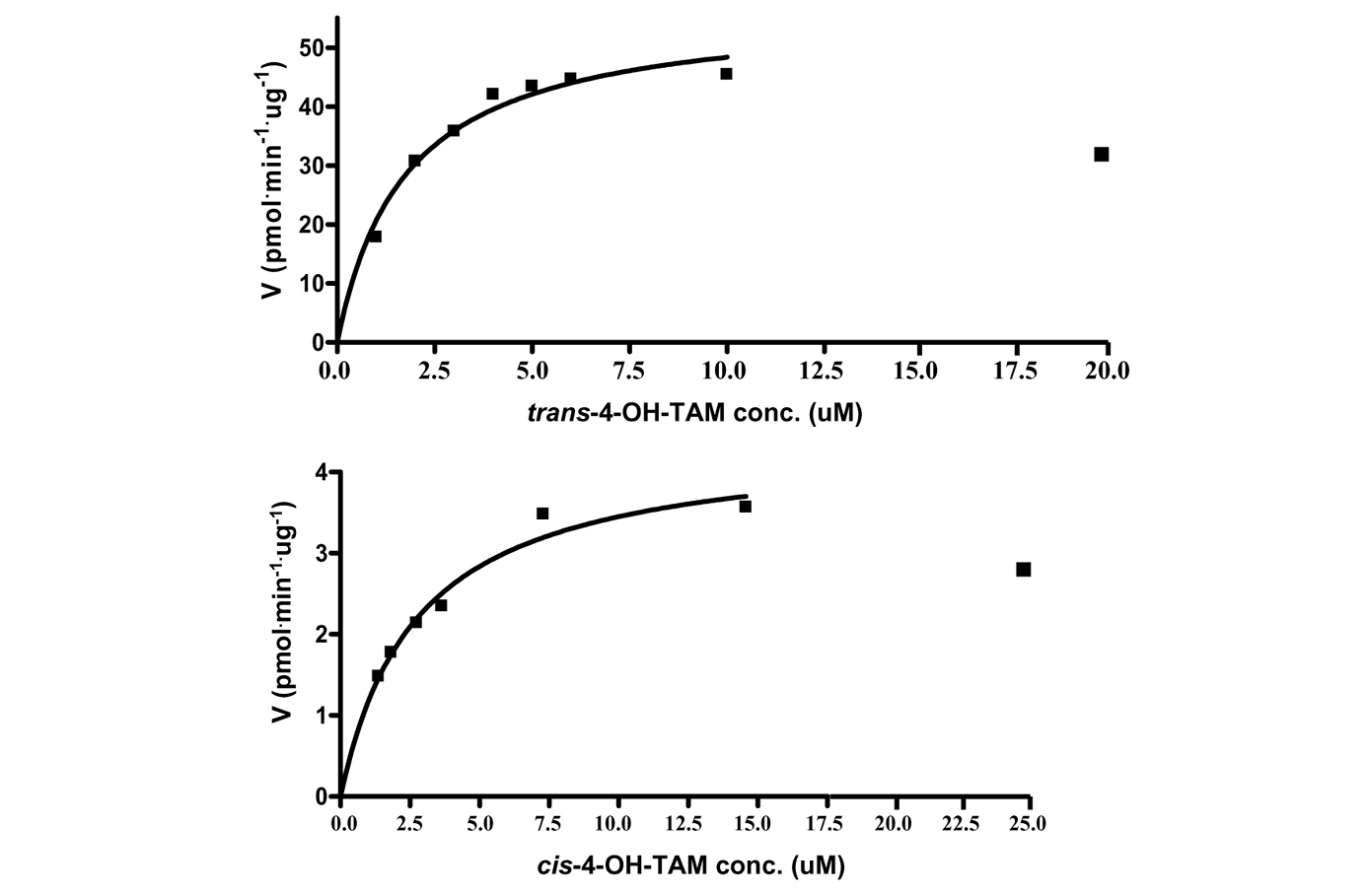
Concentration curves for 4-hydroxytamoxifen glucuronide formation with microsomes from wild-type UGT1A4-overexpressing cells. Concentration curve incubations for *trans*-4-hydroxytamoxifen (trans-4-OH-TAM) and *cis*-4-hydroxytamoxifen (*cis*-4-OH-TAM) glucuronide formation were performed for 30 min at 37°C using 10 μg microsomal protein.

As shown in a concentration curve for both isoforms of 4-OH-TAM (Figure 4), the rate of UGT1A4-catalyzed glucuronide formation was fully saturated at approximately 10–15 μM for *trans*-4-OH-TAM and *cis*-4-OH-TAM in our assay conditions. Using a concentration range of 4-OH-TAM (1–10 μM for *trans*-4-OH-TAM and 1–15 μM for *cis*-4-OH-TAM) and a specific incubation time (30 min), the apparent *K*_m _values and the *V*_max_/*K*_m _ratios for UGT1A4-induced glucuronidation of *trans*-4-OH-TAM and of *cis*-4-OH-TAM were 2.2 ± 0.4 μM and 29.3 ± 2.7 μl/min/μg, and 2.1 ± 0.4 μM and 2.0 ± 0.3 μl/min/μg, respectively (performed in three independent experiments for both isomers).

### Analysis of TAM and *trans*-4-OH-TAM, *cis*-4-OH-TAM glucuronidation by human UGT1A4 variants

**Figure 5:**
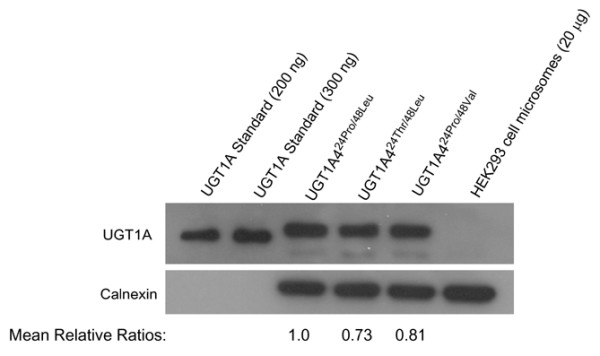
Western blot analysis of UGT1A4 protein from UGT1A4-overexpressing cell lines. Equal amounts (20 μg) of microsomal protein were loaded for each lane. The amount of UGT1A4 expression was normalized against the levels of calnexin in each lane and was quantified by densitometry relative as described in Materials and methods. Untransfected HK293 cell protein was analyzed as a negative control for UDP-glucuronosyltransferase detection.

To determine whether the Pro>Thr amino acid change at codon 24 or the Leu>Val amino acid change at codon 48 affected UGT1A4 enzyme activity against TAM or 4-OH-TAM isomers, stable HK293 cell lines overexpressing either the UGT1A4^24Thr/48Leu ^or the UGT1A4^24Pro/48Val ^variant isoforms were created by site-directed mutagenesis using the pcDNA3.1/V5-His-TOPO plasmid expressing wild-type UGT1A4^24Pro/48Leu ^as a template. Semiquantitative western blot analysis showed high levels of UGT1A4 protein in microsomes prepared from wild-type UGT1A4^24Pro/48Leu^-overexpressing and UGT1A4^24Thr/48Leu^-overexpressing or UGT1A4^24Pro/48Val^-overexpressing HK293 cell lines (Figure 5). The UGT1A4 expression levels were normalized to the levels of the endoplasmic reticulum-specific protein, calnexin, in each lane as measured by densitometry and were determined against varying amounts of human UGT1A protein (200–300 ng; also measured by densitometry). The results demonstrated that the level of expression of UGT1A4 in the UGT1A4^24Thr/48Leu^-overexpressing and UGT1A4^24Pro/48Val^-overexpressing cell lines was respectively 0.73-fold and 0.81-fold that observed in the wild-type UGT1A4-overexpressing cell line.

As shown in Table 1, kinetic analysis demonstrated that higher glucuronidation activities were observed for UGT1A4^24Pro/48Val^-overexpressing microsomes as compared with microsomes from wild-type UGT1A4^24Pro/48Leu^-overexpressing cells against TAM and against both the *trans *and *cis *isomers of 4-OH-TAM. A significantly (*P *≤ 0.02) lower *K*_m _value (~1.6-fold to 1.8-fold) was observed for both 4-OH-TAM isomers, while a near-significant (*P *= 0.053) decrease in *K*_m _was observed for TAM – for the UGT1A4^24Pro/48Val ^variant compared with wild-type UGT1A4. The *V*_max_/*K*_m _ratio for the UGT1A4^24Pro/48Val ^variant was significantly (*P *≤ 0.005) higher than that observed for the wild-type UGT1A4 isoform after normalization for UGT1A4 expression by western blotting for both the *trans *and *cis *isomers of 4-OH-TAM. No significant effect on enzyme kinetics was observed for the UGT1A4^24Thr/48Leu ^variant against either isomer of 4-OH-TAM or against TAM itself.

## Discussion

The present study is the first to examine the potential role of UGT genetic polymorphisms on the metabolism of TAM and its active metabolites. Limited studies have previously been reported identifying the UGT enzymes involved in TAM metabolism. The hepatic UGT2B15 was shown to be the major UGT active against *cis*-4-OH-TAM, while a second hepatic UGT (UGT1A4) was suggested to possess limited activity against both the *cis *and *trans *isomers of 4-OH-TAM [29]. In more recent studies [30], UGT1A4 was shown to be the only active UGT against TAM, forming a quarternary ammonium-linked glucuronide with TAM's *N*, *N*-dimethylaminoalkyl side chain. The fact that UGT1A4 was shown to be active against TAM is not surprising given that this activity is consistent with UGT1A4's activity spectrum to produce *N*-glucuronidated metabolites with other compounds [31-34,39-41].

To better characterize the glucuronide specificity of UGT1A4 against 4-OH-TAM in the present study, HPLC assays were conducted using UGT1A4-overexpressing cells. In this study, UGT1A4 exhibited significant activity with very low apparent *K*_m _values against both *trans*-4-OH-TAM and *cis*-4-OH-TAM. The single glucuronide peaks observed for incubations of UGT1A4-overexpressing microsomes with either *trans*-4-OH-TAM or *cis*-4-OH-TAM were sensitive to alkali as well as to β-glucuronidase, suggesting that these glucuronides corresponded to 4-OH-TAM-*N*^+^-glucuronide. This pattern of glucuronide formation was similar to that observed in human liver microsomes, where both the TAM-4-*O*-glucuronide and the 4-OH-TAM-*N*^+^-glucuronide were detected in incubations with either the *trans *or *cis *isomers of 4-OH-TAM, with the peaks corresponding to 4-OH-TAM-*N*^+^-glucuronide sensitive to treatment with alkali and β-glucuronidase. Both *trans*-4-OH-TAM-*N*^+^-glucuronide and *cis*-4-OH-TAM-*N*^+^-glucuronide peaks were confirmed by LC–MS to be 4-OH-TAM monoglucuronides.

These data suggest that – identical to previous observations for TAM [30], and as in recent studies by Ogura and colleagues [42] for 4-OH-TAM – UGT1A4 forms a quarternary ammonium-linked glucuronide with the *N*, *N*-dimethylaminoalkyl side chain of 4-OH-TAM. This activity is consistent with UGT1A4's activity spectrum to produce *N*-glucuronidated metabolites with other compounds [31-34,39-41].

The *K*_m _values reported in the present study for wild-type UGT1A4-induced glucuronidation of *trans*-4-OH-TAM and *cis*-4-OH-TAM were 2.2 μM and 2.1 μM, respectively. These values are 35-fold lower than that observed previously for UGT1A4 against the *trans *and *cis *isoforms of 4-OH-TAM [29]. The differences in kinetic data observed between studies for the same enzyme may be due to the fact that a baculosome overexpression system was used in the analysis of UGT1A4 in previous studies while microsomes from UGT1A4-overexpressing cells were used for the present study.

While *trans*-4-OH-TAM exhibits approximately 100 times the level of antiestrogenic activity compared with nonmetabolized tamoxifen and is probably a significant contributor to overall TAM-associated antiestrogenic activity [16-21], its desmethyl derivative, endoxifen, exhibits roughly the same antiestrogenic activity as 4-OH-TAM, but appears to be more abundant than 4-OH-TAM in the serum of TAM-treated women [19,43,44]. Studies examining the glucuronidation of endoxifen have not yet been performed, but, unlike TAM and 4-OH-TAM, the *N *position on endoxifen is demethylated, suggesting that endoxifen may perhaps be a less effective substrate for *N*-glucuronidation by enzymes such as UGT1A4. Studies aimed to identify the UGTs that are active against endoxifen are currently ongoing.

The two previously identified missense UGT1A4 polymorphisms examined in this study are located at codons 24 and 48, resulting in Pro>Thr and Leu>Val amino acid changes, respectively [35,36]. The codon 24 polymorphism, which was previously linked to increased glucuronidation activity against the tobacco-specific nitrosamine 4(methylnitrosamino)1(3pyridyl)1butanol [36] in *in vitro *assays using individual human liver microsomes, exhibited no association with altered glucuronidation capacity *in vitro *for 4-OH-TAM or for TAM in the present study. This pattern is also different from the apparent decrease in activity observed for this variant against β-naphthylamine, benzidine, *trans*-androsterone and dihydrotestosterone, although statistical analysis for glucuronidation rates between variants against each of these compounds were not provided in these studies [35]. This suggests that the functional effects of the UGT1A4 codon 24 polymorphism may be substrate dependent. Alternatively, *in vitro *functional analysis of the codon 24 polymorphism using a cell-line-overexpression system may not be optimal for studies of its effect on glucuronidation kinetics. A significant association was, however, observed for the UGT1A4 codon 48 polymorphism and 4-OH-TAM glucuronidation. The increased activity observed for the UGT1A4^24Pro/48Val ^variant as compared with the wild-type UGT1A4^24Pro/48Leu ^isoform against *trans*-4-OH-TAM, *cis*-4-OH-TAM and TAM suggests that the codon 48 polymorphism may significantly alter UGT1A4 enzymatic function against TAM and its active metabolites. As it is not yet clear whether UGT1A4 is active against endoxifen, the role of the UGT1A4^24Pro/48Val ^variant on endoxifen glucuronidation is presently not known.

Previous studies have demonstrated that variant genotypes for enzymes involved in the TAM metabolism may significantly alter overall patient response to TAM. The low-activity SULT1A1*2 allele was linked to increased rates of mortality in breast cancer patients treated with TAM [45]. In addition, the use of antidepressants, which act to inhibit CYP2D6, resulted in significantly smaller reductions in plasma hydroxylated TAM metabolite levels in TAM-treated patients with a variant CYP2D6 genotype [19,43], supporting a role for the CYP2D6 genotype in overall patient response to TAM. Variant, low-activity/expression alleles in CYP2D6, CYP2B6 and CYP2C9 were similarly correlated with levels of *trans*-4-OH-TAM formation in human liver microsomes from individual subjects [46].

The data presented in the present report. suggest that subjects with the UGT1A4^24Pro/48Val ^variant could potentially be similarly impacted with respect to individual patient response to TAM treatment and TAM-related toxicities. The prevalence of the UGT1A4^24Pro/48Val ^variant is relatively high (9.5% in a German Caucasian population [35]) so this could impact a relatively large percentage of the population given the large number of women being treated with TAM, with approximately 19% of Caucasian females expected to have at least one UGT1A4^24Pro/48Val ^variant allele. Long-term studies examining patient response to TAM and TAM-induced toxicities versus UGT1A4 genotypes will be necessary to more fully evaluate the role of UGT genotypes on TAM therapeutic efficacy.

## Conclusion

Results from this study indicate that UGT1A4 exhibits very high activity against TAM and against both isomers of 4-OH-TAM, and that the UGT1A4 codon 48 polymorphic variant is associated with altered TAM and 4-OH-TAM glucuronidation activities. These studies suggest that subjects with the UGT1A4^24Pro/48Val ^variant could potentially be similarly impacted with respect to individual patient response to TAM treatment and TAM-related toxicities.

## Abbreviations

DMEM = Dulbecco's modified Eagle's medium; HPLC = high-performance liquid chromatography; *K*_m _= Michaelis-Menten equilibrium constant; LC–MS = liquid chromatography–mass spectrometry; Leu = leucine; 4-OH-TAM = 4-hydroxytamoxifen; 4-OH-TAM-*N*^+^-glucuronide = 4-hydroxytamoxifen quaternary ammonium glucuronide; TAM = tamoxifen; TAM-*N*^+^-glucuronide = tamoxifen quaternary ammonium glucuronide; PCR = polymerase chain reaction; Pro = proline; RT = reverse transcriptase; Thr = threonine; UDPGA = UDP-glucuronic acid; UGT = UDP-glucuronosyltransferase; Val = valine.

## Competing interests

The authors declare that they have no competing interests.

## Authors' contributions

DXS designed the experimental plan, performed all kinetic analysis and contributed to tissue culture experiments for the studies described in this paper. GC helped to develop HPLC separation protocols and contributed to all data analysis and discussions. RWD guided all molecular aspects of this paper and contributed to mammalian tissue culture experiments and cell microsome preparations. KD performed the western blot analysis and helped to establish UGT1A4 cell lines. J-LF created the UGT1A4 cell lines. PL was instrumental in the planning, supervision and data analysis, and cowrote the manuscript with DXS. All authors participated in the revision of the manuscript and gave final approval of the version to be published.

## Figures and Tables

**Table 1:** Kinetic analysis of UGT1A4-induced glucuronidation of tamoxifen, *trans*-4-hydroxytamoxifen and *cis*-4-hydroxytamoxifen

Substrate	UGT1A4 variant	*V*_max _value (pmol/min/μg UGT1A4 protein)	*K*_m _value (μM)	*V*_max_/*K*_m _ratio (μl/min/μg UGT1A4 protein)
*trans*-4-Hydroxytamoxifen	UGT1A4^24Pro/48Leu^	62.4 ± 5.8	2.2 ± 0.4	29.3 ± 2.7
	UGT1A4^24Thr/48Leu^	54.9 ± 11.4	1.6 ± 0.1	33.2 ± 4.9
	UGT1A4^24Pro/48Val^	49.3 ± 2.8	1.2 ± 0.1*	40.8 ± 1.4**
*cis*-4-Hydroxytamoxifen	UGT1A4^24Pro/48Leu^	4.1 ± 0.1	2.1 + 0.4	2.0 + 0.3
	UGT1A4^24Thr/48Leu^	5.0 + 0.7	2.3 + 0.4	2.2 + 0.5
	UGT1A4^24Pro/48Val^	5.6 + 0.2	1.3 + 0.1^†^	4.4 + 0.4^‡^
Tamoxifen	UGT1A4^24Pro/48Leu^	68.0 ± 8.6	2.0 ± 0.51	35.2 ± 9.6
	UGT1A4^24Thr/48Leu^	62.1 ± 2.6	1.5 ± 0.20	41.0 ± 7.0
	UGT1A4^24Pro/48Val^	52.1 ± 10.1	1.3 ± 0.10^§^	40.1 ± 7.5

Data are expressed as the mean ± standard deviation for three independent experiments. Values are significantly (*P = 0.01, **P = 0.005, ^†^*P *= 0.02, ^‡^*P *= 0.001) or near-significantly (^§^*P *= 0.053) higher than that observed for microsomes from wild-type UGT1A4^24Pro/48Leu^-overexpressing cells.
